# Collaborative Reverse Logistics Network for Infectious Medical Waste Management during the COVID-19 Outbreak

**DOI:** 10.3390/ijerph19159735

**Published:** 2022-08-08

**Authors:** Xuan Luo, Wenzhu Liao

**Affiliations:** School of Management Science and Real Estate, Chongqing University, Chongqing 400044, China

**Keywords:** infectious medical waste, reverse logistics, collaborative, multi-participant, optimization

## Abstract

The development of COVID-19 in China has gradually become normalized; thus, the prevention and control of the pandemic has encountered new problems: the amount of infectious medical waste (IMW) has increased sharply; the location of outbreaks are highly unpredictable; and the pandemic occurs everywhere. Thus, it is vital to design an effective IMW reverse logistics network to cope with these problems. This paper firstly introduces mobile processing centers (MPCs) into an IMW reverse logistics network for resource-saving, quick response, and the sufficient capacity of processing centers. Then, a multi-participant-based (public central hospitals, disposal institutions, the logistics providers, and the government) collaborative location and a routing optimization model for IMW reverse logistics are built from an economic, environmental perspective. An augmented ε-constraint method is developed to solve this proposed model. Through a case study in Chongqing, it is found that for uncertain outbreak situations, fixed processing centers (FPCs) and MPCs can form better disposal strategies. MPC can expand the processing capacity flexibly in response to the sudden increase in IMW. The results demonstrate good performance in reduction in cost and infection risk, which could greatly support the decision making of IMW management for the government in the pandemic prevention and control.

## 1. Introduction

The COVID-19 pandemic, which started in 2019, has been devastating worldwide. Its long incubation period, high infectivity, multiple transmission routes, general population susceptibility, and long recovery period make the pandemic tricky [1]. After the infection is cured, there are more sequelae in addition to the respiratory system. The possibility of variation exists in the long-term development of the virus [2]. The pandemic is evolving globally. By December 2021, 275,701,716 cases had been confirmed abroad and 129,904 in China [3]. The World Health Organization (WHO) has raised the risk level of this outbreak to “very high”, which is the highest level [4].

Since the outbreak of COVID-19, many measures for pandemic prevention and control have been used, such as restricting the flow of people, large-scale COVID-19 testing, and vaccine research [5]. However, the pandemic has not been fully contained. People are at the stage of regularly preventing and controlling pandemics, and it will remain for a long time [6]. In response to this situation, China has proposed “six modernizations” [7]. As a result, inevitably increased infectious medical waste (IMW) is generated during the treatment of infected patients. Accordingly, many other types of IMW, including disposable personal protective equipment (PPE), such as facial masks, gloves, aprons, googles, shoes covers, in addition to diagnoses tests and vaccination sets, have increased considerably [8]. The daily average medical waste generation from May to August 2020 was approximately 69.1 tons per day in Wuhan, which was still 26% higher than the year before pandemic, due to increased COVID-19 testing and precautions and treatment that took place after the pandemic began [9]. The outbreak of COVID-19 has also impacted medical services. For example, it has postponed nonemergency care, ranging from office visits to elective surgery. In the United States, the amount of elective care has dropped significantly, and some services have decreased by 50% or more [10]. Hospitals have also invested more in equipment and procedures for pandemic prevention and control while reducing spending on other medical services [11]. To reduce the adverse impact of the sudden pandemic, the efficient management of IMW is of high concern to society at present.

### 1.1. IMW Management

IMW refers to the subset of waste generated at healthcare facilities that are unsuitable for disposal in a municipal solid waste system due to pathogenic concerns [12]. Coronaviruses can remain active on inanimate hard surfaces, such as metal, glass, or plastic for up to nine days. Contaminated IMW has thus becomes a new source of infection if left inadequately treated [13]. In particular, IMW can bring huge occupational and environmental risks, such as spreading coronavirus, and polluting land and water, when mixed into municipal solid waste [14,15]. IMW generated by the COVID-19 outbreak has posed an environmental and health concern in many countries, especially developing countries [16].

Currently, some studies have found many deficiencies in the management of IMW. The processing capacity of IMW is usually at a low level, and the management pattern is relatively extensive; most of the processing has been achieved through incineration and landfilling [17]. There is also a substantial cost when disposing of IMW. Cost is crucial in the waste management chain [18]. Many countries, therefore, do not separate IMW from medical waste, and even treat it with municipal waste. The unorganized management and collection schemes from hospitals and other sources have caused dumping and landfilling of IMW, along with the municipal solid waste [19]. In particular, in some developing countries, such as Ethiopia, only 42% of waste handlers had adequate knowledge [20]. People’s awareness of IMW processing also needs to be enhanced. After the COVID-19 outbreak, the rate of masks discarded increased two-fold. The districts that had contributed 55–60% of total mask usage had higher pollution levels [21]. The proliferation of masks is also an extremely important issue of waste management.

The current IMW management practice has achieved initial results in the management process and treatment methods. From the perspective of the management process, it includes classified collection, temporary storage, transport, and disposal [9]. Many participants are involved in the management process of IMW. The citizens and hospitals are the sources of medical waste [22]. The hospital is responsible classification, collection, and storage and bears the corresponding costs [23]. In the transportation link between each node, the third-party logistics provider bears the main costs and benefits [24]. In the final disposal link, disposal institutions with business licenses are responsible for the disposal of medical waste and obtain benefits through incineration power generation [25]. On the whole, the government departments dominate in integrated waste management plan, regulatory framework, and the construction of public infrastructure [26].

In terms of treatment methods for IMW, incineration technology develops originally as the mainstream in China, which is of course the easiest and most effective method. But there exist some other disposal techniques, such as small mobile processing facilities carrying out pyrolysis, high temperature sterilization, and steam sterilization [27]. Although the processing capacity and efficiency of these mobile facilities are not as good as that of large-scale incineration plants, they have the characteristics of greater mobility, faster response speed, and less environmental pollution, which are valuable in emergency situations [28]. For example, in the COVID-19 outbreak, the mobile processing facilities adopted in Wuhan have greatly improved the treatment efficiency [25].

### 1.2. Logistics

Research on logistics has always been the focus of academic attention. The logistics in business often focuses on transportation cost, transportation capacity, and transportation efficiency [29], such as the logistics of e-commerce and logistics enterprises [30,31]. The research on urban logistics often takes into account the factors of traffic congestion and environmental factors and promotes sustainable urban development [32].

When the pandemic started, the rapidly increasing IMW required an efficient and fast response logistics system to handle the situation. In response to this sudden natural disaster, emergency logistics were put into action [33]. Maghfiroh et al. investigated the application of the dynamic vehicle routing problem for last mile distribution during disaster response [34]. Nezhadroshan et al. designed a humanitarian logistic network with multiple central warehouses and local distribution centers and provided a plan to combat uncertainty [35]. Humanitarian logistics and emergency management put more emphasis on data availability, the dynamic nature of disasters, uncertainty, and the multiple objectives of a disaster supply chain [36].

The response to the outbreak of the pandemic required the collaboration of all parties involved, which is also a concern in logistics research. Wang et al. studied a collaborative mechanism collaborative multicenter vehicle routing problem with time windows and mixed deliveries and pickups [37]. Tavakoli et al. introduced collaboration in terms of sharing scarce resources among different facilities in a maintenance logistics network to reduce the delays in the delivery of repaired devices [38]. Hribernikab et al. provided a possible solution for last-mile deliveries through collaborations between express and parcels in city logistics [39].

The research on IMW logistics is mainly based on reverse logistics, which is defined as “the process of planning, implementing, and controlling the efficient, cost-effective flow of raw materials, in-process inventory, finished goods and related information from the point of consumption to the point of origin to recapture value or proper disposal” [40]. Qiu et al. proposed a MILP model for production routing problems with reverse logistics and remanufacturing [41]. Rahimi and Ghezavati discussed reverse logistics in the field of construction and gave a mixed-integer linear programming model to reduce environmental effects and increase social effects [42]. Gholizadeh realized scenario-based network modeling under uncertain conditions, in order to minimize the total cost of the reverse logistics network and optimize the capability of planning centers [43].

This paper focuses on the IMW reverse logistics problem under the pandemic. Existing research has provided a lot of relevant work. Budak and Ustundag considered the additional temporary disposal facilities to expand network capacity in the case of a pandemic [44]. Yu et al. constructed a mixed-integer linear programming model with three objectives: minimize the total cost, minimize the risk of transportation and processing, and maximize the amount of uncollected IMW [45]. Kargar et al. considered all the possible generation points of MW from the source of waste generation and predicted the quantity of MW generated for pandemic situations, so as to collect all IMW [46]. Eren and Rifat Tuzkaya decided on the transport routing optimization of MW, with the lowest risk and cost through the safety score of hospitals [47]. Govindan et al. considered a time window-based green vehicle routing problem and adopted the fuzzy goal programming method, which met the need for the timeliness of IMW during the pandemic [48].

However, the current COVID-19 outbreak places new demands on the logistics of IMW. The large increase in IMW has resulted in insufficient existing processing capacity. The location of outbreaks in this pandemic are highly unpredictable, which will persist for a long time [49]. According to the “Regulation on Administration of Medical Waste” [50], IMW must be stored temporarily for no more than two days [51]. Based on the principle of centralized disposal in the vicinity, all IMW should be delivered to the centralized disposal unit for rapid disposal [52]. Therefore, the logistics of IMW needs to be flexible and efficient to deal with uncertainty, timeliness, and insufficient capacity. The disposal cost of IMW is higher than the rest of waste [53], and improper disposal can lead to secondary infection [20]. Costs and risks in the link of IMW’s transportation and processing are key concerns. The logistics network design of IMW needs optimization due to its economic and environmental impact [54]. Few current studies consider these issues simultaneously.

Literature surveys show that the methods of emergency logistics can be applied to deal with uncertainty and timeliness, and the collaboration of multi-participants tend to minimize the total cost and risk and realizes the global optimization. On the other hand, the research on IMW treatment technology remains at the technical level, and how to apply the characteristics of a mobile processing facility to IMW reverse logistics has not been studied in previous research. In order to support the pandemic prevention and control in China, this paper is devoted to proposing a novel multi-participant-based (public central hospitals, disposal institutions, the logistics providers, the government) collaborative location and routing optimization model for IMW from economic and environmental perspectives. Each participant has its role to play. Public central hospitals are responsible for collecting IMW and bearing part of the cost of IMW disposal. The disposal institution refers to a separate profit-making institution in the IMW management. The third-party logistics provider is responsible for the costs and risks of the transportation process. The government formulates IMW management rules and regulations and performs supervision functions. After the outbreak of COVID-19, it is also responsible for allocating resources from all parties, implementing pandemic prevention and control, and paying for the most cost.

The innovation of this paper can be summarized as a new IMW reverse logistics network with mobile processing facilities in mobile processing centers (MPCs), which is designed to flexibly change the network layout. This network has important practical significance for pandemic prevention and control under normalized pandemics, since the high uncertainty of the outbreak location requires a reverse logistics network with faster response and lower risk.

To integrate innovative points into the actual IMW management, the main objectives of this paper are summarized as follows:

1. Build a collaborative location and routing optimization model for IMW by introducing MPCs and mobile processing facilities;
2. Develop a quantitative method for the estimation of IMW generated in the stage of a pandemic;
3. Consider infection risk and transportation timeliness to meet the requirements of the storage time restriction of IMW treatment (i.e., not exceed 48 h);
4. Provide a case study in a real situation to obtain general managerial implications.

## 2. Materials and Methods

**Figure 1:**
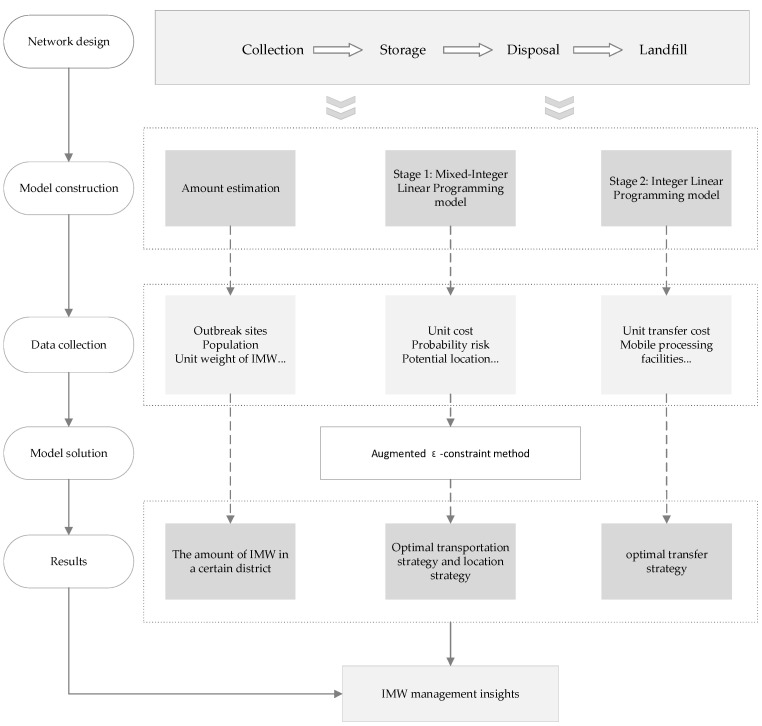
Flow diagram to show the method in this study.

In this study, the method of modeling and simulation is adopted to analyze the actual case, and it finally comes up with management suggestions for all the participants. Firstly, the reverse logistics network of IMW is redesigned. Then, the models are constructed to simulate the actual case: (1) the amount of IMW generated in all districts under different outbreak situations is estimated by a prediction model; (2) transportation and location strategies are optimized by a mixed-integer linear programming model; (3) transfer strategies are optimized by an integer linear programming model. Finally, a case in Chongqing is discussed, and the specific value of relevant parameters in the models is obtained through field research and literature research. The amount of IMW in all districts is calculated and actual strategies are optimized. Figure 1 shows the method of this paper.

### 2.1. The Structure of IMW Network

The location of a particular outbreak of the pandemic is highly unpredictable in the wider pandemic. In other words, the pandemic seems controllable in a small area, but difficult to be eliminated in a large area. Once a local case breaks out in a certain district, small-scale outbreaks will occur around the district if the initial emergency response is not carried out. After some small-scale pandemics have been effectively controlled, other communities may be in the stage of development. Hence, on the whole, a regular pandemic shows a situation “increasing here and decreasing there”, which means that the pandemic and various prevention and control measures will exist for a long time. For this prevention and control requirement, the IMW management logistics should also be adjusted accordingly.

**Figure 2:**
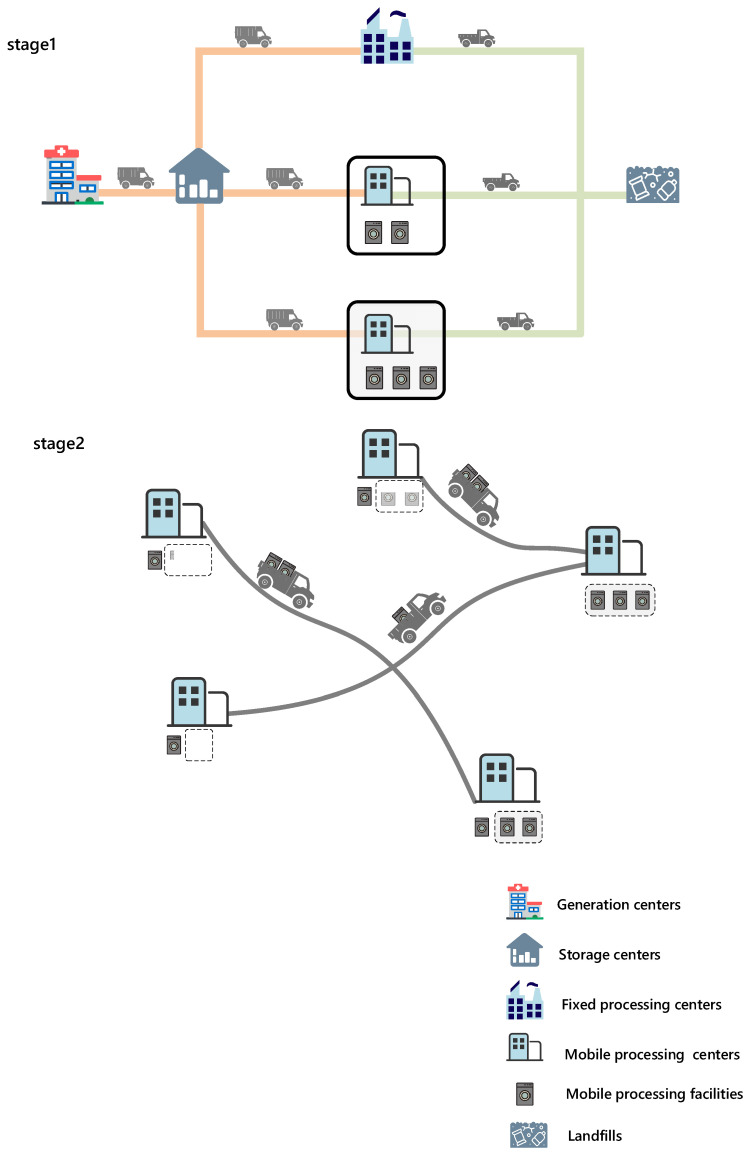
The structure of the proposed network.

Therefore, in response to the regular COVID-19 outbreak, this paper designed the structure of IMW reverse logistics network presented in Figure 2.

The first stage in Figure 2 shows the hierarchy and the nodes of the network. IMW generation centers, storage centers, FPCs, MPCs, mobile processing facilities, and landfills are involved in the network. They break down into three categories, consisting of generation, storage, and treatment of IMW. The generation part is comprised of existing hospitals, temporary hospitals, clinics, laboratories, and residential areas generating a different amount of IMW in terms of an outbreak situation [46]. All the IMW is transported to the second part—storage centers—so that IMW can be well restrained from spreading viruses. Within the 48 h storage time limit, IMW needs to be promptly sent to the third part—treatment part—for disinfection and sterilization. The nodes of this part are divided into FPC, in which processing facilities are not movable, and MPC, which accommodates a different number of mobile processing facilities. Most IMW is processed by incineration in FPC, while incineration disposal vehicle, movable steam, and microwave sterilization equipment tend to work in MPC dealing with the remaining IMW.

The second stage in Figure 2 demonstrates the role of MPCs and mobile processing facilities. According to uncertain outbreak sites, mobile processing facilities will be transferred from one MPC to another. The small and mobile emergency processing facilities, as dynamic processing centers, can minimize the risk of virus transmission during transportation process and increase the flexibility of processing centers.

In this study, there are some assumptions given as below.

1. IMW originates from existing hospitals, temporary hospitals, clinics, laboratories, and residential areas.
2. IMW has been collected from all generators to the largest public hospital of the district, so the network starts with the largest public hospitals from all districts.
3. The processing capacity of FPC is definite. In addition, FPCs and MPCs both have partial storage capability, which is also definite.
4. The processing capacity of MPC is proportional to the number of mobile processing facilities. One MPC can accommodate a definite number of mobile processing facilities.
5. To improve efficiency, all IMW are transported to the storage centers with no storage in IMW generation centers.

### 2.2. Model Construction

The routing optimization model for IMW reverse logistics is built by involving mixed integer linear programming model, which has been used widely for the optimization of complex systems, such as those arising in biology, medicine, transportation, telecommunications, sports, and national security [55]. In this study, different reverse logistics schemes can be formed, according to actual outbreak situation. The whole scheme is divided into two stages.

1. First stage: produces demand distribution of different mobile devices.

In this stage, with the aim of minimizing the total cost and risk in IMW management, the following issues are determined:

- The location of storage centers, MPCs, and FPCs;
- The demand flow speed between each center;
- The number of mobile processing facilities in each MPC.

2. Second stage: generates corresponding mobile scheme, according to demand distribution in different outbreak situations.

In this stage, with the aim of minimizing the total cost of transfer, the following issues are determined:

- The number of mobile processing facilities transferred between MPCs;
- The path of transferring mobile processing facilities.

In the first stage, this paper is devoted to constructing a routing optimization model for IMW management: collection, transportation, storage, treatment, and final disposal.

The notation is given below.
**Indices***c*IMW generation center*s*IMW storage center*i*IMW FPC*j*IMW MPC*t*outbreaks at different times**Parameters**$GCW_{c}^{t}$the amount of IMW produced by IMW generation center *c* in the time *t*$FC_{i}$fixed operating cost for FPC *i*$MC_{j}$fixed operating cost for MPC *j*$SC_{S}$fixed operating cost for storage center *s*$FPC_{i}$unit processing cost for FPC *i*$MPC_{j}$unit processing cost for MPC *j*$SSC_{s}$unit processing cost for storage center *s*DICcost of disassembly and installation for each mobile processing facilityPRCprocurement cost for each mobile processing facilitymtcunit transportation cost using IMW transfer vehicletfcunit transportation cost for transferring a mobile processing facility$DCS_{cs}$distance from IMW generation center c to storage center *s*$DSI_{si}$distance from storage center *s* to FPC *i*$DSJ_{sj}$distance from storage center *s* to MPC *j*$DJ{J^{\prime }}_{jj^{\prime }}$distance from MPC *j’* to MPC *j*$SCA_{s}$maximum capacity of storage center *s*$FCA_{i}$maximum capacity of FPC *i*MCAmaximum capacity of each mobile processing facilityMZmaximum number of mobile processing facility accommodated in each MPC *j*$PbA_{s}$risk of infection around storage centers$PbA_{i}$risk of infection around FPCs$PbA_{j}$risk of infection around MPCs$PbA_{t}$risk of infection during transportation$PopS_{cs}$population exposure on the route from IMW generation center *c* to storage center *s*$PopI_{si}$population exposure on the route from storage center *s* to FPC *i*$PopJ_{sj}$population exposure on the route from storage center *s* to MPC *j*$PopS_{s}$population exposure around storage center *s*$PopI_{i}$population exposure around FPC *i*$PopJ_{j}$population exposure around MPC *j*βupper limit for the storage time of IMW in the storage center**Decision Variables**$X_{j}^{t}$0/1 variable for selection of MPC *j* in the time *t*$Y_{i}^{t}$0/1 variable for selection of FPC *i* in the time *t*$W_{s}^{t}$0/1 variable for selection of storage center *s* in the time *t*$Z_{j}^{t}$the number of mobile processing facilities in the MPC *j* in the time *t*$ZN_{j}^{t}$the number of newly acquired mobile processing facilities in MPC *j* in the time *t*$ZT_{jj^{\prime }}^{t}$the number of mobile processing facilities that move from MPC *j* to MPC *j’* at the end of the time *t*$VS_{cs}^{t}$the flow rate of IMW transferred from IMW generation center *c* to storage center *s* in the time *t*$VI_{si}^{t}$the flow rate of IMW transferred from storage center *s* to FPC *i* in the time *t*$VJ_{sj}^{t}$the flow rate of IMW transferred from storage center *s* to MPC *j* in the time *t*

With the aim to minimize the total cost consisting of fixed cost, operation cost, storage cost, and transportation cost, $Z_{1}$, denoted as the total cost, is formulated in Equation (1). In addition, with the aim to minimizing the total risk in the process of storage, treatment and transportation, $Z_{2},$ denoted as the total risk, is formulated in Equation (2).

(1) $$\begin{array}{ll} Z_{1}=\underset{i}{\sum }FC_{i}\times Y_{i}^{t} & +\underset{j}{\sum }MC_{j}\times X_{j}^{t}+\underset{s}{\sum }SC_{s}\times W_{s}^{t}+\underset{i}{\sum }\underset{s}{\sum }FPC_{i}\times VI_{si}^{t}+\underset{j}{\sum }\underset{s}{\sum }MPC_{j}\times VJ_{sj}^{t}\;\\ & +\underset{s}{\sum }SSC_{s}\times \left(\underset{c}{\sum }VS_{cs}^{t}-\underset{i}{\sum }VI_{si}^{t}-\underset{j}{\sum }VJ_{sj}^{t}\right)+\underset{s}{\sum }\underset{c}{\sum }mtc\times DCS_{cs}\times VS_{cs}^{t}\\ & +\underset{i}{\sum }\underset{s}{\sum }mtc\times DSI_{si}\times VI_{si}^{t}+\underset{j}{\sum }\underset{s}{\sum }mtc\times DSJ_{sj}\times VJ_{sj}^{t}\; \end{array}$$

In Equation (1), $\sum_{i}FC_{i}\times Y_{i}^{t}$ denotes the fixed operating cost for FPCs. $\sum_{j}MC_{j}\times X_{j}^{t}$ denotes the operating cost for MPCs. $\sum_{s}SC_{s}\times W_{s}^{t}$ denotes the fixed operating cost for storage centers. $\sum_{i}\sum_{s}FPC_{i}\times VI_{si}^{t}$ denotes the variable cost of treating IMW at FPCs. $\sum_{j}\sum_{s}MPC_{j}\times VJ_{sj}^{t}$ denotes the variable cost of treating IMW at MPCs. $\sum_{s}SSC_{s}\times \left(\sum_{c}VS_{cs}^{t}-\sum_{i}VI_{si}^{t}-\sum_{j}VJ_{sj}^{t}\right)$ denotes the storage cost at storage centers. $\sum_{s}\sum_{c}mtc\times DCS_{cs}\times VS_{cs}^{t}$ denotes the transportation cost of transporting IMW from the point of generation to storage centers. $\sum_{i}\sum_{s}mtc\times DSI_{si}\times VI_{si}^{t}$ denotes the transportation cost of transporting IMW from storage centers to FPCs. $\sum_{j}\sum_{s}mtc\times DSJ_{sj}\times VJ_{sj}^{t}$ denotes the transportation cost of transporting IMW from storage centers to mobile treatment centers.

(2) $$\begin{array}{ll} Z_{2}=\;\underset{S}{\sum }PbA_{s}\times & PopS_{s}\times \left(\underset{c}{\sum }VS_{cs}^{t}-\underset{i}{\sum }VI_{si}^{t}-\underset{j}{\sum }VJ_{sj}^{t}\right)+\underset{i}{\sum }\underset{s}{\sum }PbA_{i}\times PopI_{i}\times VI_{si}^{t}\\ & +\underset{j}{\sum }\underset{s}{\sum }PbA_{j}\times PopJ_{j}\times VJ_{sj}^{t}+\underset{s}{\sum }\underset{c}{\sum }PbA_{t}\times PopS_{cs}\times VS_{cs}^{t}\;+\underset{i}{\sum }\underset{s}{\sum }PbA_{t}\times PopI_{si}\times VI_{si}^{t}\\ & +\underset{j}{\sum }\underset{s}{\sum }PbA_{t}\times PopJ_{cs}\times VJ_{sj}^{t} \end{array}$$

In Equation (2), $\sum_{S}PbA_{s}\times PopS_{s}\times \left(\sum_{c}VS_{cs}^{t}-\sum_{i}VI_{si}^{t}-\sum_{j}VJ_{sj}^{t}\right)$ denotes the storage risk of storage centers storing IMW. $\sum_{i}\sum_{s}PbA_{i}\times PopI_{i}\times VI_{si}^{t}$ denotes the processing risks of FPCs dealing with IMW. $\sum_{j}\sum_{s}PbA_{j}\times PopJ_{j}\times VJ_{sj}^{t}$ denotes the processing risks of MPCs dealing with IMW. $\sum_{s}\sum_{c}PbA_{t}\times PopS_{cs}\times VS_{cs}^{t}$ denotes the transport risk of IMW when it is transferred from the point of generation to storage centers. $\sum_{i}\sum_{s}PbA_{t}\times PopI_{si}\times VI_{si}^{t}$ denotes the transport risk of IMW when it is transferred from storage centers to FPCs. In addition, $\sum_{j}\sum_{s}PbA_{t}\times PopJ_{cs}\times VJ_{sj}^{t}$ denotes the transport risk of IMW when it is transferred from storage centers to MPCs.

In the second stage on Figure 2, the object of transportation is changed to mobile processing facilities. The amount of mobile processing facilities for each MPC in the first stage has been determined. At this stage, there is no longer any risk of infection, and only the cost of transportation is considered.

(3) $$Z_{3}=\underset{j}{\sum }PRC\times ZN_{j}^{t}+\frac{1}{2}\underset{j}{\sum }\underset{j^{\prime }}{\sum }DJ{J^{\prime }}_{jj^{\prime }}\times DIC\times \left|ZT_{jj^{\prime }}^{t}\right|\;$$

Equation (3) means the total cost of transporting mobile processing facilities at the second stage, which can be viewed as an integer programming model.

Hence, the mathematical model is presented as


$$Min\;\left(Z_{1},Z_{2},Z_{3}\right)$$

s.t.

(4) $$GCW_{c}^{t}=24\times \underset{s}{\sum }VS_{cs}^{t},\;\forall c,t\;$$

(5) $$\underset{c}{\sum }VS_{cs}^{t}-\underset{i}{\sum }VI_{si}^{t}-\underset{j}{\sum }VJ_{sj}^{t}\geq 0,\;\forall s,t\;$$

(6) $$24\times \beta \left(\underset{c}{\sum }VS_{cs}^{t}-\underset{i}{\sum }VI_{si}^{t}-\underset{j}{\sum }VJ_{sj}^{t}\right)\leq SCA_{s}\times W_{s}^{t},\;\forall s,t\;$$

(7) $$24\times \underset{s}{\sum }VI_{si}^{t}\leq FCA_{i}\times Y_{i}^{t},\;\forall i,t\;$$

(8) $$24\times \underset{s}{\sum }VJ_{sj}^{t}\leq MCA\times X_{j}^{t}\times Z_{j}^{t},\;\forall j,t\;$$

(9) $$Z_{j}^{t}\leq MZ,\;\forall j,t\;$$

(10) $$Z_{j}^{t}=\underset{j^{\prime }}{\sum }ZT_{j^{\prime }j}^{t}+ZN_{j}^{t}+Z_{j}^{t-1},\;\forall j,t\;$$

(11) $$ZT_{j^{\prime }j}^{t}+ZT_{jj^{\prime }}^{t}=0,\;\forall j^{\prime },j\;$$

(12) $$X_{j}^{t},Y_{i}^{t},W_{s}^{t}\in \left\{0,1\right\},\;\forall i,j,s,t\;$$

(13) $$Z_{j}^{t},ZN_{j}^{t},ZT_{jj^{\prime }}^{t}\in \left\{0,1,2,\ldots ,MZ\right\},\;\forall j,j^{\prime },t\;$$

(14) $$VS_{cs}^{t},VI_{si}^{t},VJ_{sj}^{t}\geq 0,\;\forall c,s,i,j,t\;$$

Equation (4) balances the flow between IMW generation centers and storage centers. Equation (5) balances the flow between storage centers and FPCs and MPCs. Equations (6)–(8) guarantee that the inflow of waste to storage centers, the inflow of waste to FPCs, and the inflow of waste to MPCs are no more than their capacity. Equation (9) ensures the number of mobile processing facilities in MPCs does not exceed its capacity. Equations (10) and (11) balance the mobile processing facilities in the second stage when a mobile processing facility is transferred. Equations (12)–(14) show the characteristics of these decision variables.

### 2.3. Model Solution

#### 2.3.1. Amount Estimation

The estimation and prediction of IMW quantity can improve IMW management, which facilitates managers to make decisions in advance and respond to emergencies more quickly. When a pandemic breaks out in a district, the IMW will increase suddenly because of the prevention and control measures. The estimation of the amount of IMW during the pandemic can be divided into two parts:

1. The amount of basic IMW before the outbreak;
2. The steep increase in the number of protective suits for health workers, COVID-19 tests, vaccination, and treatment of infected patients after the outbreak.

The related parameters are given as follows.
**Parameters**
ddistrict where IMW generation center *c* is located$Pop_{d}$total population in district *d*$PopM_{d}$total population of medical staff in district *d*$PopC_{d}$the number of confirmed cases in district *d*$W_{PPE}$average PPE’ s weight per worker$W_{test}$the weight of items for one COVID-19 test$W_{MW}$the weight of basic IMW, such as masks per person, before outbreak$W_{cure}$the weight of IMW produced by treating an infected patient$R_{PPE}$acceptance rate of PPE before outbreak$RO_{PPE}$acceptance rate of PPE after outbreak$R_{test}$acceptance rate of COVID-19 tests before outbreak$RO_{test}$acceptance rate of COVID-19 tests after outbreak$B_{d}^{t}$1 if district *d* breaks out the pandemic, 0 otherwise

Hence, the quantity of IMW in a certain area are calculated as

(15) $$\begin{array}{ll} GCW_{c}^{t}=W_{MW}\times & Pop_{d}+W_{PPE}\times PopM_{d}\times \left(R_{PPE}+B_{d}^{t}\times RO_{PPE}\right)+W_{test}\times Pop_{d}\times \left(R_{test}+B_{d}^{t}\times RO_{test}\right)\\ & +W_{cure}\times PopC_{d}\times B_{d}^{t} \end{array}$$

In Equation (15), $GCW_{c}^{t}$ is divided into four items: $W_{MW}\times Pop_{d}$ denotes basic IMW, such as masks for daily work of prevention and control. $W_{PPE}\times PopM_{d}\times \left(R_{PPE}+B_{d}^{t}\times RO_{PPE}\right)$ denotes the weight of protective clothing for medical staff; there are great differences between the start and end of the outbreak. $W_{test}\times Pop_{d}\times \left(R_{test}+B_{d}^{t}\times RO_{test}\right)$ denotes the weight of IMW produced by nucleic acid test. $W_{cure}\times PopC_{d}\times B_{d}^{t}$ denotes the weight of IMW produced by treating a confirmed case with COVID-19.

#### 2.3.2. Linearization

The proposed model has a non-linear term due to the multiplication of a positive integer variable by a binary variable. Hence, auxiliary variables and adding additional constraints are introduced to linearize objective function [56]. This paper gives a new auxiliary variable, shown as

(16) $$L_{j}^{t}=X_{j}^{t}\times Z_{j}^{t}\;\forall j,t\;$$

where $X_{j}^{t}$ is a binary variable, and $Z_{j}^{t}$ is an integer variable.

The non-linear constraint of the model is replaced by the following constraints:

(17) $$L_{j}^{t}\geq Z_{j}^{t}-M\times \left(1-X_{j}^{t}\;\right)\;\forall j,t\;$$

(18) $$L_{j}^{t}\leq Z_{j}^{t}+M\times \left(1-X_{j}^{t}\;\right)\;\forall j,t\;$$

(19) $$L_{j}^{t}\leq M\times X_{j}^{t}\;\forall j,t\;$$

(20) $$L_{j}^{t}\geq 0\;and\;integer\;\forall j,t\;$$

where *M* is a large positive number.

#### 2.3.3. Augmented ε-Constraint Method

To balance the relationship between cost and risk, this proposed model can be viewed as a multi-objective mixed-integer linear programming model. One of features in such multi-objective problems is a series of Pareto optimal solutions. Haimes firstly proposed a method based on ε-constraint to solve these bi-objective problems and showed its good performance [57]. Based on this, many pieces of research have been done. However, these traditional ε-constraint approaches might produce weakly non-dominated solutions; the augmented ε-constraint method is thus developed, which adopts slack/surplus variables to standardize original inequality constraints as equality ones, and augments the original objectives with these additional variables multiplied by a small positive number [58].

The original ε-constraint method is described as:

(21) $$\min\tilde{z}\left(x\right)=\;z_{1}\left(x\right)\;$$

(22) $$s.t.\;z_{2}\left(x\right)\leq e_{2}\;$$

(23) x∈X 

For this proposed optimization model, the total cost objective in Equation (1) is retained and the value level, $e_{2},$ is introduced to restrain the total risk objective. A slack variable, $\mu_{2},$ is set to turn the inequality constraint on the total risk objective into an equality constraint. Moreover, in order to avoid the scaling problems, the slack variable, $\mu_{2},$ is further divided by the range $v_{2}=z_{2}^{max}-z_{2}^{min}$ of the total risk objective. Then, this proposed model can be improved, given as

(24) $$\min\tilde{z}\left(x\right)=\;z_{1}\left(x\right)-\rho \mu_{2}/v_{2}\;$$

(25) $$s.t.\;z_{2}\left(x\right)+\mu_{2}=e_{2}\;$$

(26) $$\mu_{2}\geq 0\;$$

(27) x∈X 

If $v_{2}$ is divided into g equal intervals, g + 1 well dispersed non-dominated solutions can be obtained. Then, the weight between the two goals can be adjusted by the size of $e_{2}$.

(28) $$e_{2}=z_{2}^{max}-kv_{2}/g\;$$

(29) k=1,2,3,…,g 

where the greater *k* is, the greater the weight of risk will be. It should be noted that these weights are determined based on real cases, which is a key issue for the decision-maker.

### 2.4. Data Collection

Chongqing is the largest city in western China, as well as the most populous city in China [59], thus pandemic prevention and control becomes very important to this city. In this city, there are 38 districts and counties and 3 development zones [60].

#### 2.4.1. Data on Locations

According to Baidu map searching and an official query website—National Health Commission of the People’s Republic of China [61]—each district in Chongqing has the largest central public hospital. To simplify the research, these central public hospitals are taken as the initial 41 IMW generation centers, as shown in Table 1. The potential locations of storage centers, FPCs, and MPCs, and were acquired from the issuance of hazardous waste business licenses on the official website of Chongqing Ministry of Ecological Environment [62]. Actually, except for storage centers with only storage capabilities, we use FPCs and MPCs as the remaining storage centers due to their storage function. All FPCs and MPCs are located based on existing waste disposal sites with IMW business licenses and industrial parks with construction conditions of waste disposal sites. The sites for the FPCs are existing IMW waste disposal sites with a processing capacity of 5000 kg per day. The sites of MPCs follows the principle of wide coverage and small processing capacity. The existing waste disposal sites with a processing capacity of 5000 kg per day and some industrial parks are selected.

**Figure 3:**
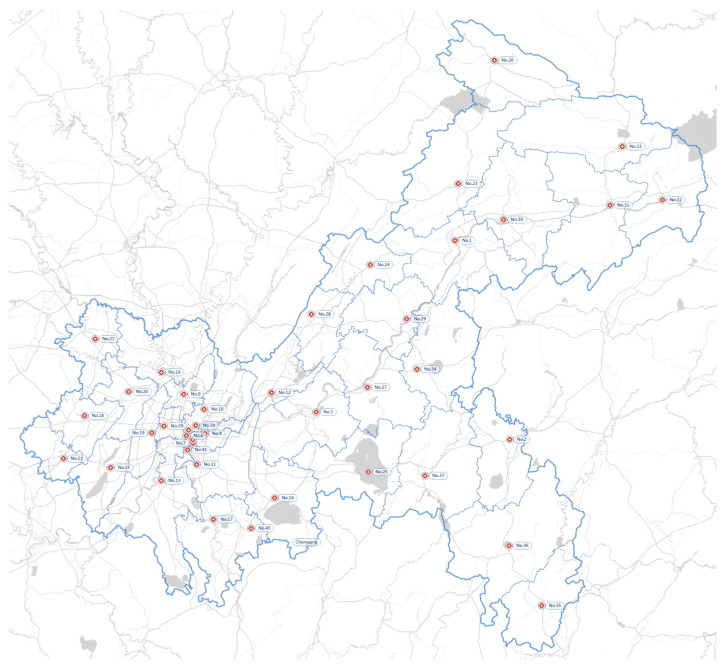
Generation centers of IMW in Chongqing.

**Figure 4:**
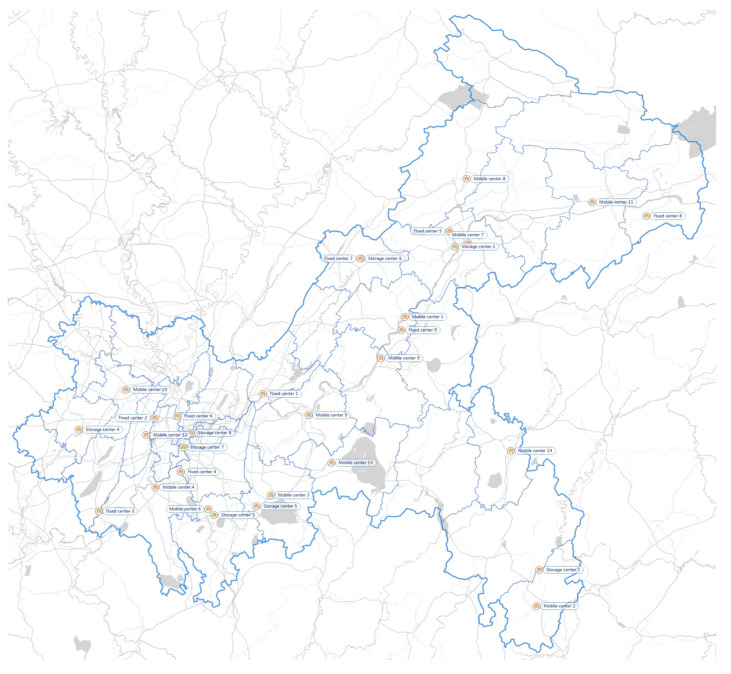
Storage centers in Chongqing.

**Figure 5:**
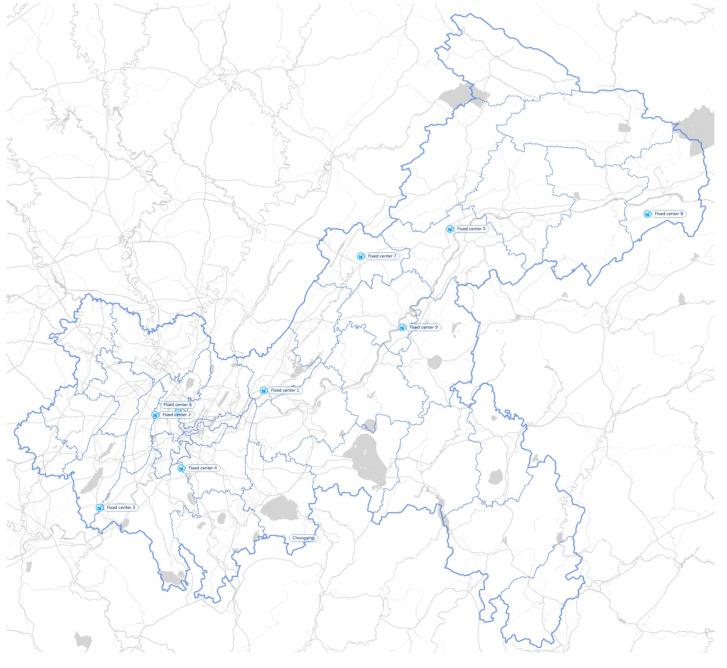
FPCs in Chongqing.

**Figure 6:**
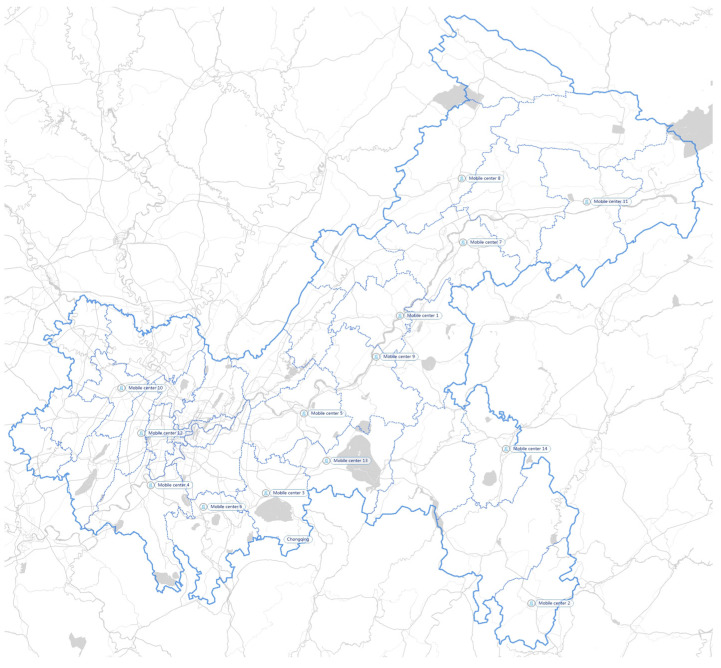
MPCs in Chongqing.

As mentioned above, there are 41 generation points in the network. In addition, 9 FPCs, 14 MPCs, and 31 storage centers are identified across the area of Chongqing. Each district has its corresponding medical institutions and IMW disposal system. Figure 3 shows the location of 41 representative generation centers distributed in 41 areas. Figure 4 illustrates the location of 31 existing storage centers. Figure 5 illustrates the location of 9 FPCs. Figure 6 illustrates the candidate location of 14 MPCs.

#### 2.4.2. Data on Estimation of IMW’s Generation

The parameters, such as the total population, the number of medical staff, population density, etc., were obtained from the statistical yearbooks of Chongqing [63], shown in Table 1. During the pandemic, it is assumed that each person generates 2 g IMW per day, due to prevention and control measures [2]. The amount of basic IMW (kg/d), such as masks, can be calculated by (total population in a district) × 2.

The PPE’s weight, nucleic acid testing reagent’s weight, and the amount of IMW generated by treating a COVID-19 patient were identified from the statistics collected in other pieces of research [8]. Specifically, the first item—PPE for healthcare workers in healthcare facilities, including surgical face mask, face mask N-95, face shield, white suit, gown, goggle glass, gloves, and shoes cover—totals up to 550 g every worker per day [64]. Usually, the second item—a set of COVID-19 tests—is composed of nasopharyngeal swab and a bottle of a chemical preservative, with a total weight of 12.53 g [65]. As for the third item, The Asian Development Bank proposed 3.4 kg/d IMW generated by an infected person [66].

To show the change in the weight of IMW before and after the outbreak, the acceptance rate is set to represent the proportion of people using COVID-19 protection measures in the total population. By assuming a pre-outbreak reception rate of 10% and a post-outbreak reception rate of 70%, daily PPE’s weight (kg) per worker can be calculated by (average PPE’s weight per worker) × (total number of workers) × (reception rate). Based on the actual situation, a reception rate of 1% before an outbreak and 50% after an outbreak is assumed. Hence, total weight generated from the COVID-19 diagnostic tests can be calculated by (weight of items for one test) × (total population) × (reception rate) [67]. Since the number of infected people tends to be controlled at a certain number, this study set the number of infected people at the outbreak site to be 1000. Hence, the amount of IMW (kg/d) from treatments can be calculated by (number of infected persons) × 3.4.

#### 2.4.3. Data on Risks

The setting of the risk value is based on the risk of the transportation and treatment of hazardous waste [68]. The storage risk of a storage center $PbA_{s}$ is set as 0.0003, while the processing risk of FPC and MPC (i.e., $PbA_{i}$ and $PbA_{j}$) are both 0.0007. The accident rate of a truck in transit is 0.4 × 10^−6^/km, and the truck accident release probability is 0.9. In the selection of infection radius, the infectious radius of FPC is obviously larger than that of MPC with smaller scale. According to the policy “Classified Catalogue of Medical Waste”, when the storage temperature is ≥5 °C, the storage shall not exceed 24 h, and if the storage temperature is <5 °C, the storage shall not exceed 72 h. Therefore, storage centers are often equipped with special cold storage to store IMW that has not been processed in time [69]. As the IMW is often stored in a closed cold storage or a closed warehouse, while it may be exposed for a while when being processed, the infection radius is smaller. Therefore, this paper assumes that the infection radius of FPC is 3 km, and that of MPC and storage center is 2 km [68]. The number of exposures can be calculated by Equation (30).

(30) $$PopS_{s},PopI_{i},PopJ_{j}=\pi r^{2}\left(m^{2}\right)\times population\;desity\left(\mathrm{people}/m^{2}\right)\;$$

And transport risk can be calculated by Equation (31)

(31) $$PbA_{t}=\left(0.4\times 10^{-6}/\mathrm{km}\times 0.9\right)\times travel\;length\left(\mathrm{km}\right)\;$$

Finally, the total risk probability is calculated by (the amount of IMW) × (the number of people exposed).

#### 2.4.4. Data on the Rest Parameters

In this paper, the decision variable is flow rate, namely the volume of transportation per hour, so the capacity of processing centers and related costs are in days. The parameters related to the cost and capacity in the first stage are shown in Table 2 and Table 3, who are the most probable values in the actual situation. In the second stage, this paper set the transport cost of each mobile processing facility to be 400 yuan/km, and the price of purchasing a new facility is 24,000 yuan, according to the survey.

## 3. Results and Discussions

**Figure 7:**
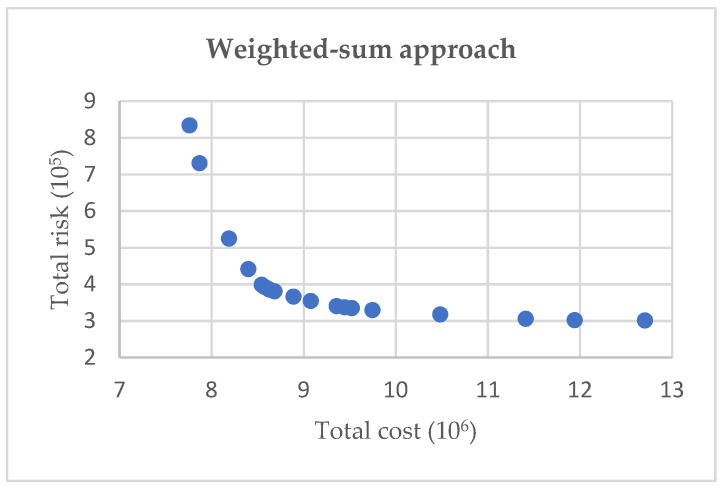
Solutions in weighted-sum approach.

**Figure 8:**
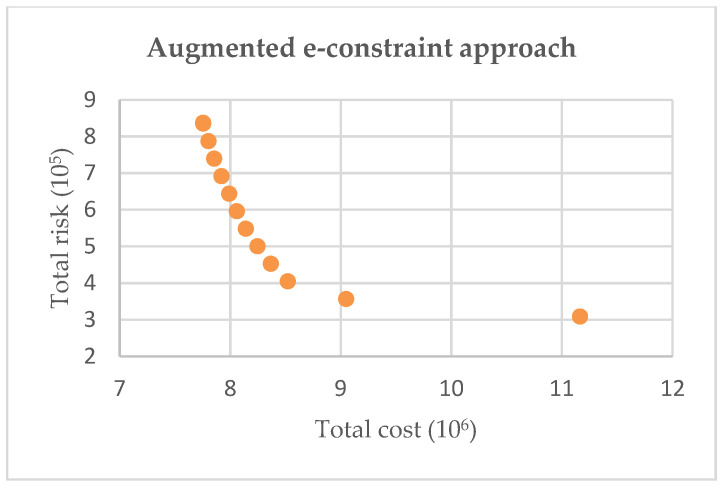
Solutions in augmented ε-constrained approach.

To choose a more appropriate solution, two generation methods were compared: conventional weighted-sum method and augmented ε-constraint method. In order to discuss the effectiveness and efficiency, 20 bounded non-dominated solutions were generated in the case of no outbreak sites, illustrated in Figure 7 and Figure 8. It is obvious that the augmented ε-constraint approach can generate better frontiers under different upper numbers of solutions by distributing the solutions as uniformly as possible. Hence, these 20 solutions can be well configured for IMW management system and provide sufficient reference for decision-makers.

In order to show the analysis, the subsequent discussion with breakout points uses the 11th solution calculated under ε-constraint approach as an example, where the weight of cost and risk target accounts for 0.55 and 0.45, respectively.

To reflect the actual situation of the pandemic, this paper assumes the situations of two kinds of different outbreak sites. When t = 1, there is no COVID-19 outbreak; When t = 2, the COVID-19 outbreak occurs in the west of Chongqing—Dazu, Tongliang, Bishan, Yongchuan and Shapingba (the outbreak may start from the border of Chongqing and spread inward); When t = 3, the outbreak occurs in the northeast of Chongqing—Wanzhou, Kaizhou, Chengkou, and Wuxi. The amount of IMW from each of the above-mentioned districts is presented in Table 4. In addition, the total amount of IMW has increased dramatically after the COVID-19 pandemic. The results of the new open centers in every situation are illustrated in Table 5. The value of the objectives is illustrated in Table 6.

In the absence of a single site outbreak, namely t = 1, 5 FPCs and 15 storage centers are opened, but no MPCs required, which is presented in Table 5. When there is no outbreak, the processing capacity of FPC is sufficient.

When t = 2, the total amount of IMW in Chongqing would increase by 110.97%. This sudden increase of 10 tons of IMW would be transferred to MPCs for processing. If cost is taken as the only target, the total cost increases by 112.41%, compared to before the outbreak. At this time, all FPCs are required to be open to reduce the cost. On the other hand, if risk is taken as the only target, the value increases seven times, compared with that before the outbreak, which will produce a higher risk of infection affecting people’s lives and health. The 6 FPCs and 29 storage centers with all MPCs are suggested to be opened to diversify the risk. Finally, if both the cost and risk are considered simultaneously, the cost increases by 0.97%, while the risk decreases by 16.77%. At this solution, 21 storage centers are opened, with all FPCs and 4 MPCs, each of which accommodates three mobile processing facilities, which reaches the upper limit. Once an MPC is opened, it will accommodate the mobile processing facilities as much as possible. This will not add more operating costs.

When t = 3, the total amount of IMW in Chongqing would nearly double. The location of new MPCs has changed from MPC 4, 6 to MPC 7, 8. MPC 1, 7, 8 accommodate three mobile processing facilities, respectively, with two in MPC 2. Because the total amount of IMW is a little less, there is no need for so many mobile processing facilities.

For comparison, this paper calculates the result without applying MPC at t = 3, only utilizing existing high-capacity FPCs for IMW processing. The result is shown in Table 7 that the final cost increases by 0.08%, total risk increases by 9.66%, and all FPCs and all storage centers are put into use. It can be seen that the application of MPCs is of great benefit to the reduction in risks and costs.

In the second stage, it is assumed that each MPC has a mobile processing facility when there is no outbreak. When the outbreak occurs in the west, MPC 5, 7, 8, 9, 10, 11, 12, 14 deploys a device to MPC 4, 2, 4, 1, 6, 6, 2, 1, respectively. No additional new facilities are required. Then, in the case of another outbreak in the east, one device should be transferred from MPC 2 to 7, two more devices should be purchased and installed in MPC 7, and three devices should be transferred from MPC 4 to 8 to complete the emergency response. The scheme is illustrated in Table 8.

Without the mobile processing facilities in MPC, all the processing facilities would be repurchased. In the first period, eight new units should be added, and in the second period six new units need to be purchased. The total cost is more than three times higher. The change of cost is presented in Table 9. The cost of transporting a mobile processing facility is much lower than the cost of purchasing a new fixed processing facility when the transportation distance is not too far. Therefore, mobile processing facilities can greatly improve the utilization rate of facilities and reduce the cost of using facilities.

It is found that the storage center is used to share the treatment and transportation risks. FPC is mainly used to deal with most IMW and reduce the total cost of the network, while MPC is devoted to dealing with the parts that FPCs cannot handle, responding to emergencies, and sharing the risk of infection. The utilization rate of the processing centers in the middle of Chongqing is higher than that at the edge of the city. However, due to the large flow of people in the middle of Chongqing, it is prone to infection risk. Mobile processing facilities in MPCs are often transferred across the districts to maximize the utilization of existing facilities.

Affected by the transfer cost and transfer efficiency, the transfer process can be carried out when the transfer distance is relatively close. If the transfer cost exceeds the new acquisition cost, the purchase of new equipment will be better.

### 3.1. Sensitivity Analysis

#### 3.1.1. For Outbreak Sites

**Figure 9:**
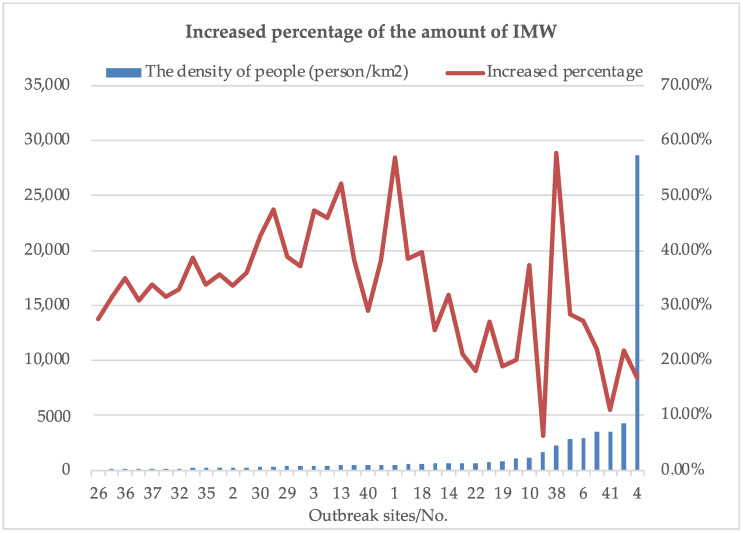
Effects of different outbreak sites (sorted according to population density) on the amount of IMW.

**Figure 10:**
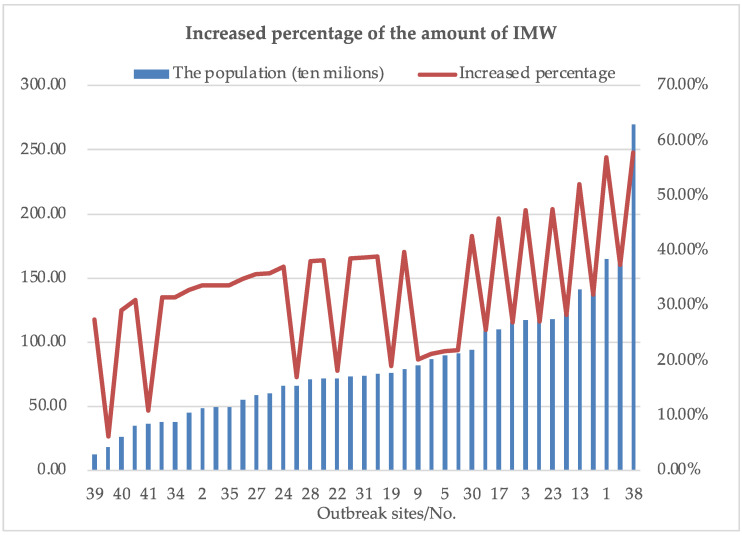
Effects of different outbreak sites (sorted according to population) on the amount of IMW.

One of the most influential decision factors that have significant effects on the amount of IMW is the outbreak site. The great differences are considered in total population and population density between these 41 districts. Accordingly, districts with high population will generate more IMW, while ones with high population density will have a greater risk of infection. Therefore, 41 IMW generation points are sorted according to total population and population density, respectively. A comparison between the proportion of the increase in the amount of IMW shows that the total population has a greater effect on the amount of IMW. The results are presented in Figure 9 and Figure 10. It can be concluded that the COVID-19 outbreak site No.38 should be focused and controlled because of its high risk of infection caused by its large population and high population density.

#### 3.1.2. For Capacity and Cost of Mobile Processing Facility

The parameters of MPCs would make a direct effect on final solutions. For example, the capacity of a mobile processing facility affects the demand of mobile processing facilities, and then their procurement cost would affect the strategy of transportation. Facility with higher capacity is usually more expensive. Therefore, five cases with different capacity and procurement cost are discussed, shown in Table 10. It is seen that, increasing the capacity of a mobile processing facility would increase the total cost in stage 1 and decrease the total risk slightly. However, there is an anomaly in the trend of cost in stage 2, due to reducing a new device.

In general, parameters of a mobile processing facility have a greater impact in stage 2. The total cost in stage 2 decreases as the procurement cost and capacity increase, so they have an adverse correlation.

#### 3.1.3. For Time Limit

Although the 48 h time limit is specified in current regulations, it may be changed along with new quarantine regulations. In this paper, β is defined as the time limit in storage centers. Different β are taken into account to observe the impact of these changes on total cost and risk.

**Figure 11:**
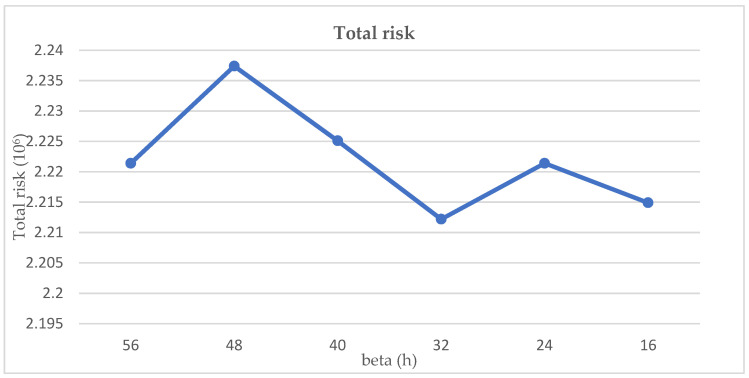
Sensitivity analysis of total risk regarding the changes in time limit.

**Figure 12:**
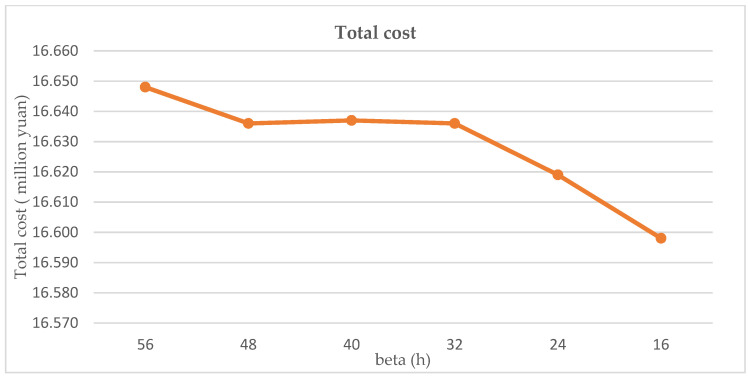
Sensitivity analysis of total cost regarding the changes in time limit.

It can be seen that in Figure 11 and Figure 12, the change of time limit has a direct correlation with total cost and risk. The total cost decreases as β decreases because the cost of storage decreases more than the cost of treatment increases. However, the total risk fluctuates as β decreases, which means the risk and β are not positively correlated.

Through the case study discussed above, some recommendations are provided to support the construction of the reverse logistics network of IMW for the multi-participants from the management perspective, concluded as follows:

1. The various branches of government need to work together: The public construction department should fully consider the location and function of the processing centers in combination with the actual situation when planning. The financial department should give financial and policy support to the businesses that produce and sell small and mobile processing facilities. The supervision department should supervise the management and treatment of IMW by hospitals and processing institutions, avoiding them disregarding risks for reducing costs. The pandemic prevention and control department should flexibly adjust prevention and control policies, focusing on areas with large populations and high population density.
2. The public hospitals are the source of IMW generation and bear the costs and risks in the collection and storage process. Timeliness can greatly reduce the risk of infection. The storage time limit can be set more flexibly. If the storage cost is relatively low, the storage time could be appropriately extended to reduce the total cost. If the processing cost is relatively low, the storage time could be appropriately shortened to reduce the total risk. The public hospitals can flexibly adjust the time limit according to the actual situation of the pandemic to balance the costs and risks.
3. Small and mobile processing facilities can greatly respond to emergencies. IMW can be managed more efficiently. The disposal institution can develop new businesses, build MPCs with smaller unit scale and wider distribution, and purchase mobile processing facilities for decentralized processing. Since the capacity and cost of mobile processing facilities have a greater impact on the total cost of the network, disposal institutions should choose mobile processing facilities with appropriate capacity according to their own business conditions and pandemic situation. Multiple disposal institutions can also collaborate to flexibly move facilities based on actual outbreaks to improve facility utilization.
4. In addition to just-in-time transportation of IMW, the third-party logistics providers can also operate the transfer business of mobile processing facilities. Some third-party logistics providers can even combine transshipment and processing operations to operate mobile IMW incinerators. In these transportation links, the choice of the route is the key to saving costs, and the third-party logistics provider should choose the globally optimal route.

## 4. Conclusions

The novel coronavirus has been in existence for more than two years and has spread globally, severely affecting economic development and social life. Due to the outbreak of COVID-19, the management of IMW needs to be improved to adapt to the current situation of the pandemic. IMW carries a high concentration of viruses and has a high risk of secondary transmission. Hence, it is vital to handle IMW under a prescribed process and time limit. From the perspective of current requirements of pandemic prevention and control, every link of IMW management is quite important. Since the sites of a pandemic outbreak are highly unpredictable, the amount of existing IMW treatment centers is insufficient to deal with it; however, at the same time, too many new temporary treatment centers being built could be a huge waste of resources. Therefore, this paper introduces MPC to transform existing small-scale processing centers into MPCs to cope with a sudden increase in IMW. A multi-objective model is constructed to minimize the total cost and infection risk of IMW reverse logistics network, in which the capacity of processing centers becomes a decision variable to clarify the processing needs under different outbreak scenarios. An augmented ε-constraint method is then developed to obtain a series of Pareto solutions for the decision making of IMW reverse logistics network.

Through the case study in Chongqing, the result shows that if IMW are treated centrally to reduce the total cost and the amount of waste stored, the risk would be higher. However, if IMW are processed in scattered processing centers, the overall risk would be greatly reduced while the cost would be higher, so the MPCs should be utilized to share the risk. Hence, to reconcile the contradiction between them, some corresponding policy recommendations are provided in this paper.

In future works, the research can be studied to work out how to use big data to predict the amount of IMW and how to identify the factors that have an impact on the amount of IMW from massive data. Further consideration can also be given to a low-carbon network design of IMW logistics.

## Figures and Tables

**Table 1:** Population-related parameters in 41 districts of Chongqing.

No.	Generation Center	Population $\left(\times 10^{4}\right)$	Population Density (People/km^2^)	Medical Staff (People)
1	Wanzhou	165	477.8	11,429
2	Qianjiang	49	205	3359
3	Fuling	117	397.8	8104
4	Yuzhong	66	28,695.7	4572
5	Jiangbei	90	4306.2	6234
6	Shapingba	117	2954.5	8104
7	Jiulongpo	123	2853.8	8520
8	Nanan	91	3475.2	6303
9	Beibei	82	1091.9	5680
10	Yubei	168	1153.1	11,637
11	Banan	109	597.9	7550
12	Changshou	87	612.2	6026
13	Jiangjing	141	438.4	9767
14	Hechuan	141	601.8	9767
15	Yongchuan	116	734.6	8035
16	Nanchuan	60	231.7	4156
17	Qijiang	110	400.4	7619
18	Dazu	79	550.9	5472
19	Bishan	76	830.6	5264
20	TongLiang	73	544.4	5057
21	Tongnan	72	454.3	4987
22	Rongchang	72	668.5	4987
23	Kaizhou	118	297.7	8174
24	Liangping	66	349.6	4572
25	Wulong	35	121	2424
26	Chengkou	18	54.7	1247
27	Fengdu	59	203.5	4087
28	Dianjiang	71	468	4918
29	Zhong	75	342.9	5195
30	Yunyang	94	258.5	6511
31	Fengjie	74	180.6	5126
32	Wushan	45	152.3	3117
33	Wuxi	38	94.6	2632
34	Shizhu	38	126.1	2632
35	XiuShan	49	199.8	3394
36	YouYang	55	106.4	3810
37	Pengshui	49	125.7	3394
38	Liangjiang	270	2250.0	18,702
39	High-tech	13	1689.2	866
40	Wansheng	26	459.4	1801
41	Dadukou	36	3495.1	2494

**Table 2:** Cost related parameters.

	Operating Cost (Yuan/d)	Processing Cost (Yuan/kg)	Transportation Cost (Yuan/km·kg)
FPC	70	1	0.35
MPC	50	2	
Storage center	20	0.5	

**Table 3:** Capacity parameters.

No.	Processing Capacity (kg/d)	Storage Capacity (kg/d)
Fixed center1	30,000	5000
Fixed center2	30,000	5000
Fixed center3	10,000	5000
Fixed center4	30,000	5000
Fixed center5	10,000	5000
Fixed center6	20,000	5000
Fixed center7	10,000	5000
Fixed center8	10,000	5000
Fixed center9	10,000	5000
Mobile center1	1500 × Z1	2000
Mobile center2	1500 × Z2	2000
Mobile center3	1500 × Z3	2000
Mobile center4	1500 × Z4	2000
Mobile center5	1500 × Z5	2000
Mobile center6	1500 × Z6	2000
Mobile center7	1500 × Z7	2000
Mobile center8	1500 × Z8	2000
Mobile center9	1500 × Z9	2000
Mobile center10	1500 × Z10	2000
Mobile center11	1500 × Z11	2000
Mobile center12	1500 × Z12	2000
Mobile center13	1500 × Z13	2000
Mobile center14	1500 × Z14	2000
Storage center1		4000
Storage center2		4000
Storage center3		4000
Storage center4		4000
Storage center5		4000
Storage center6		4000
Storage center7		4000
Storage center8		4000

**Table 4:** The amount of IMW from every district in different outbreak situations.

No.	Generation Center	The Amount of IMW (kg/d)		
		t = 1	t = 2	t = 3
1	Wanzhou	4341.10	4341.10	37,519.92
2	Qianjiang	1276.02	1276.02	1276.02
3	Fuling	3078.24	3078.24	3078.24
4	Yuzhong	1736.44	1736.44	1736.44
5	Jiangbei	2367.87	2367.87	2367.87
6	Shapingba	3078.24	27,594.13	3078.24
7	Jiulongpo	3236.09	3236.09	3236.09
8	Nanan	2394.18	2394.18	2394.18
9	Beibei	2157.40	2157.40	2157.40
10	Yubei	4420.03	4420.03	4420.03
11	Banan	2867.76	2867.76	2867.76
12	Changshou	2288.94	2288.94	2288.94
13	Jiangjing	3709.67	3709.67	3709.67
14	Hechuan	3709.67	3709.67	3709.67
15	Yongchuan	3051.93	27,387.34	3051.93
16	Nanchuan	1578.58	1578.58	1578.58
17	Qijiang	2894.07	2894.07	2894.07
18	Dazu	2078.47	19,736.20	2078.47
19	Bishan	1999.54	19,115.84	1999.54
20	TongLiang	1920.61	18,495.48	1920.61
21	Tongnan	1894.30	1894.30	1894.30
22	Rongchang	1894.30	1894.30	1894.30
23	Kaizhou	3104.55	3104.55	27,800.91
24	Liangping	1736.44	1736.44	1736.44
25	Wulong	920.84	920.84	920.84
26	Chengkou	473.57	473.57	7122.17
27	Fengdu	1552.27	1552.27	1552.27
28	Dianjiang	1867.99	1867.99	1867.99
29	Zhong	1973.23	1973.23	1973.23
30	Yunyang	2473.11	2473.11	22,838.02
31	Fengjie	1946.92	1946.92	1946.92
32	Wushan	1183.94	1183.94	1183.94
33	Wuxi	999.77	999.77	11,257.92
34	Shizhu	999.77	999.77	999.77
35	XiuShan	1289.18	1289.18	1289.18
36	YouYang	1447.03	1447.03	1447.03
37	Pengshui	1289.18	1289.18	1289.18
38	Liangjiang	7103.62	7103.62	7103.62
39	High-tech	328.87	328.87	328.87
40	Wansheng	684.05	684.05	684.05
41	Dadukou	947.15	947.15	947.15
Total		90,294.93	190,495.15	185,441.77

**Table 5:** Location selection.

t	Outbreak Sites	Storage Centers	FPCs	MPCs (the Amount of Mobile Processing Facilities)
1	Sites No. 0	1, 2, 4, 6, 7, 13, 15, 16, 19, 22, 23, 26, 28, 30, 31	1, 4, 7, 8, 9	
2	Sites No. 6, 15, 18, 19, 20	1, 2, 4, 6, 7, 8, 10, 11, 13, 14, 15, 16, 19, 20, 22, 23, 26, 28, 29, 30, 31	1, 2, 3, 4, 5, 6, 7, 8, 9	1 (3), 2 (3), 4 (3), 6 (3)
3	Sites No. 1, 23, 26, 30, 33	1, 2, 4, 6, 7, 8, 10, 11, 13, 14, 15, 16, 19, 20, 22, 23, 26, 28, 29, 30, 31	1, 2, 3, 4, 5, 6, 7, 8, 9	1 (3), 2 (2), 7 (3), 8 (3)

**Table 6:** Value of objectives.

**t**	Single Objective				Multi-Objective	
	z1min (Million Yuan)	z1max (Million Yuan)	z2min $\left(\times 10^{6}\right)$	z2max $\left(\times 10^{6}\right)$	z1 (Million Yuan)	z2 $\left(\times 10^{6}\right)$
1	7.7559	1.2006	0.14115	1.2174		
2	16.476	22.237	1.1231	2.6882	16.636	2.2374
3	15.985	23.997	1.0041	2.7628	16.165	2.2496

**Table 7:** Value of objectives without applying MPC.

**If No MPC**	Single Objective				Multi-Objective	
**t**	z1min (Million Yuan)	z1max (Million Yuan)	z2min $\left(\times 10^{6}\right)$	z2max $\left(\times 10^{6}\right)$	z1 (Million Yuan)	z2 $\left(\times 10^{6}\right)$
3	15.943	21.515	2.1061	2.6217	16.178	2.4670

**Table 8:** Mobile processing facility transfer scheme in different outbreak situations.

**MPC**	**The ** **Demand ** **of Mobile ** **Processing Facilities ** **in MPC**				
	**t = 1**	**The Number and Path of Trans** **fer**	**t = 2**	**the Number and Path of Transfer**	**t = 3**
1	1		3		3
2	1		3	1facility→7	2
3	1		1		1
4	1		3	3facilities→8	0
5	1	1facility→4	0		0
6	1		3		0
7	1	1facility→2	0	adding 2 facilities	3
8	1	1facility→4	0		3
9	1	1facility→1	0		0
10	1	1facility→6	0		0
11	1	1facility→6	0		0
12	1	1facility→2	0		0
13	1		0		0
14	1	1facility→1	1		1

**Table 9:** The transfer cost at second stage with or without mobile processing facility.

Stage 2	z3 (Thousand Yuan)	
	Mobile Processing Facilities	No Mobile Processing Facilities
t = 1~t = 2	564.12	1920
t = 2~t = 3	502.16	1440
Total	1066.28	3360

**Table 10:** Sensitivity analysis for MCA and PRC.

MCA	PRC (Million Yuan)	Sites No. 6, 15, 18, 19, 20		z3 (Million Yuan)		New Devices
		z1 (Million Yuan)	z2 $\left(\times 10^{6}\right)$	Distribution 1–2	Distribution 2–3	
1600	0.16	16.611	2.2879	0.6267	0.7018	2
1800	0.18	16.634	2.2422	0.5368	0.4834	1
2000	0.2	16.636	2.2374	0.4851	0.5483	2
2200	0.22	16.65	2.2085	0.4851	0.4248	0
2400	0.24	16.656	2.1957	0.4023	0.4248	0

## Data Availability

Not applicable.

## References

[1] Hao X., Cheng S., Wu D., Wu T., Lin X., Wang C. (2020). Reconstruction of the full transmission dynamics of COVID-19 in Wuhan. Nature.

[2] Adeloye D., Elneima O., Daines L., Poinasamy K., Quint J.K., Walker S., Brightling C.E., Siddiqui S., Hurst J.R., Chalmers J.D. (2021). The long-term sequelae of COVID-19: An international consensus on research priorities for patients with pre-existing and new-onset airways disease. Lancet Respir. Med..

[3] WHO Coronavirus Disease (COVID-19) Weekly Epidemiological Update and Weekly Operational Update. https://www.who.int/emergencies/diseases/novel-coronavirus-2019/situation-reports/.

[4] WHO (2021). WHO COVID-19 Dashboard. https://covid19.who.int.

[5] Kristoffersen A.E., van der Werf E.T., Stub T., Musial F., Wider B., Jong M.C., Wode K., Danell J.-A.B., Busch M., Hoenders H.R. (2021). Consultations with health care providers and use of self-management strategies for prevention and treatment of COVID-19 related symptoms. A population based cross-sectional study in Norway, Sweden and the Netherlands. Complement. Ther. Med..

[6] Yang W.Z. (2020). Thoughts on the outbreak phases of the COVID-19 changed from emergency response to the combination of emergent response and regular prevention and control activities. Zhonghua Liu Xing Bing Xue Za Zhi.

[7] Central People’s Government of the People’s Republic of China Guiding Opinions of the State Council’s Joint Prevention and Control Mechanism for Novel Coronavirus Pneumonia Epidemic. http://www.gov.cn/zhengce/content/2020-05/08/content_5509896.htm.

[8] Al-Omran K., Khan E., Ali N., Bilal M. (2021). Estimation of COVID-19 generated medical waste in the Kingdom of Bahrain. Sci. Total Environ..

[9] Chen C., Chen J., Fang R., Ye F., Yang Z., Wang Z., Shi F., Tan W. (2021). What medical waste management system may cope With COVID-19 pandemic: Lessons from Wuhan. Resour. Conserv. Recycl..

[10] Cutler D.M., Nikpay S., Huckman R.S. (2020). The Business of Medicine in the Era of COVID-19. JAMA.

[11] Chang W.-H. (2020). The influences of the COVID-19 pandemic on medical service behaviors. Taiwan J. Obstet. Gynecol..

[12] EPA Medical Waste. https://www.epa.gov/rcra/medical-waste.

[13] Kampf G., Todt D., Pfaender S., Steinmann E. (2020). Persistence of coronaviruses on inanimate surfaces and their inactivation with biocidal agents. J. Hosp. Infect..

[14] Saadat S., Rawtani D., Hussain C.M. (2020). Environmental perspective of COVID-19. Sci. Total Environ..

[15] Sangkham S. (2020). Face mask and medical waste disposal during the novel COVID-19 pandemic in Asia. Case Stud. Chem. Environ. Eng..

[16] Mol M.P.G., Caldas S. (2020). Can the human coronavirus epidemic also spread through solid waste?. Waste Manag. Res..

[17] Windfeld E.S., Brooks M.S.-L. (2015). Medical waste management—A review. J. Environ. Manag..

[18] Bening C.R., Kahlert S., Asiedu E. (2021). The true cost of solving the plastic waste challenge in developing countries: The case of Ghana. J. Clean. Prod..

[19] Thind P.S., Sareen A., Singh D.D., Singh S., John S. (2021). Compromising situation of India’s bio-medical waste incineration units during pandemic outbreak of COVID-19: Associated environmental-health impacts and mitigation measures. Environ. Pollut..

[20] Lemma H., Asefa L., Gemeda T., Dhengesu D. (2022). Infectious medical waste management during the COVID-19 pandemic in public hospitals of West Guji zone, southern Ethiopia. Clin. Epidemiol. Glob. Health.

[21] Singh D., Aryan Y., Chavan D., Tembhare M., Dikshit A.K., Kumar S. (2022). Mask consumption and biomedical waste generation rate during COVID-19 pandemic: A case study of central India. Environ. Res..

[22] Shukor F.S.A., Mohammed A.H., Sani S.I.A., Awang M. A review on the success factors for community participation in solid waste management. Proceedings of the International Conference on Management.

[23] Bamakan S.M.H., Malekinejad P., Ziaeian M. (2022). Towards blockchain-based hospital waste management systems; applications and future trends. J. Clean. Prod..

[24] Eydi A., Rastgar S. (2021). A DEA model with dual-role factors and fuzzy data for selecting third-party reverse logistics provider, case study: Hospital waste collection. Ain Shams Eng. J..

[25] Zhao H., Liu H., Wei G., Zhang N., Qiao H., Gong Y., Yu X., Zhou J., Wu Y. (2021). A review on emergency disposal and management of medical waste during the COVID-19 pandemic in China. Sci. Total Environ..

[26] Fernando R.L.S. (2018). Solid waste management of local governments in the Western Province of Sri Lanka: An implementation analysis. Waste Manag..

[27] Dharmaraj S., Ashokkumar V., Pandiyan R., Munawaroh H.S.H., Chew K.W., Chen W.-H., Ngamcharussrivichai C. (2021). Pyrolysis: An effective technique for degradation of COVID-19 medical wastes. Chemosphere.

[28] UN Environment Program (UNEP) Practical Guidelines for the Handling, Storage and Disposal of COVID-19 Infected Wastes, Including Personnel Protective Equipment. [Guidelines]. https://www.humanitarianlibrary.org/sites/default/files/2020/06/UNEP_PRACTICAL%20GUIDELINE%20FOR%20COVID%2019%20WASTE%20MANAGEMENT%20UNEP-GSC-DOS.pdf.

[29] Wang M., Wood L.C., Wang B. (2022). Transportation capacity shortage influence on logistics performance: Evidence from the driver shortage. Heliyon.

[30] Kang X., Pang H. (2022). City selection for fresh produce e-commerce’s market entry strategy: Based on the perspective of urban logistics competitiveness. Transp. Res. Interdiscip. Perspect..

[31] Wang F., Yang X., Zhuo X., Xiong M. (2019). Joint logistics and financial services by a 3PL firm: Effects of risk preference and demand volatility. Transp. Res. Part E Logist. Transp. Rev..

[32] Rześny-Cieplińska J., Szmelter-Jarosz A., Moslem S. (2021). Priority-based stakeholders analysis in the view of sustainable city logistics: Evidence for Tricity, Poland. Sustain. Cities Soc..

[33] Kundu T., Sheu J.-B., Kuo H.-T. (2022). Emergency logistics management—Review and propositions for future research. Transp. Res. Part E Logist. Transp. Rev..

[34] Maghfiroh M.F., Hanaoka S. (2018). Dynamic truck and trailer routing problem for last mile distribution in disaster response. J. Humanit. Logist. Supply Chain Manag..

[35] Nezhadroshan A.M., Fathollahi-Fard A.M., Hajiaghaei-Keshteli M. (2020). A scenario-based possibilistic-stochastic programming approach to address resilient humanitarian logistics considering travel time and resilience levels of facilities. Int. J. Syst. Sci. Oper. Logist..

[36] Yáñez-Sandivari L., Cortés C.E., Rey P.A. (2020). Humanitarian logistics and emergencies management: New perspectives to a sociotechnical problem and its optimization approach management. Int. J. Disaster Risk Reduct..

[37] Wang Y., Ran L., Guan X., Fan J., Sun Y., Wang H. (2022). Collaborative multicenter vehicle routing problem with time windows and mixed deliveries and pickups. Expert Syst. Appl..

[38] Kafiabad S.T., Zanjani M.K., Nourelfath M. (2021). Robust collaborative maintenance logistics network design and planning. Int. J. Prod. Econ..

[39] Hribernik M., Zero K., Kummer S., Herold D.M. (2020). City logistics: Towards a blockchain decision framework for collaborative parcel deliveries in micro-hubs. Transp. Res. Interdiscip. Perspect..

[40] Rogers D.S., Tibben-Lembke R.S. (1999). Going Backwards: Reverse Logistics Trends and Practices.

[41] Qiu Y., Ni M., Wang L., Li Q., Fang X., Pardalos P.M. (2018). Production routing problems with reverse logistics and remanufacturing. Transp. Res. Part E Logist. Transp. Rev..

[42] Rahimi M., Ghezavati V. (2018). Sustainable multi-period reverse logistics network design and planning under uncertainty utilizing conditional value at risk (CVaR) for recycling construction and demolition waste. J. Clean. Prod..

[43] Govindan K., Gholizadeh H. (2021). Robust network design for sustainable-resilient reverse logistics network using big data: A case study of end-of-life vehicles. Transp. Res. Part E Logist. Transp. Rev..

[44] Budak A., Ustundag A. (2016). Reverse logistics optimisation for waste collection and disposal in health institutions: The case of Turkey. Int. J. Logist. Res. Appl..

[45] Yu H., Sun X., Solvang W.D., Zhao X. (2020). Reverse Logistics Network Design for Effective Management of Medical Waste in Epidemic Outbreaks: Insights from the Coronavirus Disease 2019 (COVID-19) Outbreak in Wuhan (China). Int. J. Environ. Res. Public Health.

[46] Kargar S., Pourmehdi M., Paydar M.M. (2020). Reverse logistics network design for medical waste management in the epidemic outbreak of the novel coronavirus (COVID-19). Sci. Total Environ..

[47] Eren E., Tuzkaya U.R. (2021). Safe distance-based vehicle routing problem: Medical waste collection case study in COVID-19 pandemic. Comput. Ind. Eng..

[48] Govindan K., Nasr A.K., Mostafazadeh P., Mina H. (2021). Medical waste management during coronavirus disease 2019 (COVID-19) outbreak: A mathematical programming model. Comput. Ind. Eng..

[49] Fu Y.-L., Liang K.-C. (2020). Fuzzy logic programming and adaptable design of medical products for the COVID-19 anti-epidemic normalization. Comput. Methods Programs Biomed..

[50] P.R.C Regulation on Administration of Medical Waste. http://www.gov.cn/gongbao/content/2011/content_1860802.htm.

[51] Yong Z., Gang X., Guanxing W., Tao Z., Dawei J. (2009). Medical waste management in China: A case study of Nanjing. Waste Manag..

[52] Insa E., Zamorano M., López R. (2010). Critical review of medical waste legislation in Spain. Resour. Conserv. Recycl..

[53] Lee B.-K., Ellenbecker M.J., Moure-Ersaso R. (2004). Alternatives for treatment and disposal cost reduction of regulated medical wastes. Waste Manag..

[54] He Z.-G., Li Q., Fang J. (2016). The Solutions and Recommendations for Logistics Problems in the Collection of Medical Waste in China. Procedia Environ. Sci..

[55] Smith J.C., Taskin Z.C. (2008). Tutorial Guide to Mixed-Integer Programming Models and Solution Techniques. Optim. Med. Biol..

[56] Aalaei A., Davoudpour H. (2016). Revised multi-choice goal programming for incorporated dynamic virtual cellular manufacturing into supply chain management: A case study. Eng. Appl. Artif. Intell..

[57] Haimes Y.Y., Lasdon L.S., Wismer D.A. (1971). On a Bicriterion Formulation of the Problems of Integrated System Identification and System Optimization. IEEE Trans. Syst. Man Cybern..

[58] Mavrotas G., Florios K. (2013). An improved version of the augmented ε-constraint method (AUGMECON2) for finding the exact pareto set in multi-objective integer programming problems. Appl. Math. Comput..

[59] Bao H.X.H., Li L., Lizieri C. (2019). City profile: Chongqing (1997–2017). CITIES.

[60] Zhuang T., Qian Q.K., Visscher H.J., Elsinga M.G. (2020). An analysis of urban renewal decision-making in China from the perspective of transaction costs theory: The case of Chongqing. Neth. J. Hous. Built Environ..

[61] National Health Commission of the People’s Republic of China National Medical Institution Inquiry. http://zgcx.nhc.gov.cn:9090/unit.

[62] Chongqing Municipal Bureau of Ecology, People’s Republic of China The Issuance of Hazardous Waste Business Licenses in Chongqing. http://sthjj.cq.gov.cn/zwgk_249/gszx/wxfwjyxkzgs/202110/t20211015_9809345.html.

[63] Chongqing Municipal Bureau of Statistics of the People’s Republic of China Chongqing Statistical Yearbook Calendar Year Download (In Chinese). http://tjj.cq.gov.cn/zwgk_233/tjnj/202012/t20201214_8606164.html.

[64] Purnomo C.W., Kurniawan W., Aziz M. (2021). Technological review on thermochemical conversion of COVID-19-related medical wastes. Resour. Conserv. Recycl..

[65] Celis J.E., Espejo W., Paredes-Osses E., Contreras S.A., Chiang G., Bahamonde P. (2020). Plastic residues produced with confirmatory testing for COVID-19: Classification, quantification, fate, and impacts on human health. Sci. Total Environ..

[66] ADB Managing Infectious Medical Waste during the COVID-19 Pandemic.2. https://www.adb.org/publications/managing-medical-waste-covid19.

[67] Korkut E.N. (2018). Estimations and analysis of medical waste amounts in the city of Istanbul and proposing a new approach for the estimation of future medical waste amounts. Waste Manag..

[68] Zhao J., Huang L., Lee D.-H., Peng Q. (2016). Improved approaches to the network design problem in regional hazardous waste management systems. Transp. Res. Part E Logist. Transp. Rev..

[69] Chen Y., Guo C. (2020). Handbook of Emergency Disposal and Management of Medical Waste in China.

